# Impact of Media Reports and Environmental Pollution on Health and Health Expenditure Efficiency

**DOI:** 10.3390/healthcare7040144

**Published:** 2019-11-13

**Authors:** Ying Li, Yung-Ho Chiu, Huaming Chen, Tai-Yu Lin

**Affiliations:** 1 Business School, Sichuan University, Chengdu 610064, China; liyinggs@scu.edu.cn; 2 Department of Economics, Soochow University, Taipei 1004, Taiwan; echiu@scu.edu.tw (Y.-H.C.); eickyla@gmail.com (T.-Y.L.); 3 College of Literature and Journalism, Sichuan University, Chengdu 610064, China

**Keywords:** EBM two stage model, energy efficiency, environmental efficiency, media coverage, public health

## Abstract

Over the past few decades, China’s rapid economic, energy, and industrial developments have caused serious environmental damage. However, as there are large resource, energy use, economic, and environmental damage differences across Chinese regions, the Chinese government is seeking to reduce city pollution across the country. Most previous analyses have only looked at these issues on a single level; for example, the impact of environmental pollution on health, or energy and environmental efficiency analyses, but there have been few studies that have conducted overall analyses. Further, many of the methods that have been used in previous research have employed one-stage radial or non-radial analyses without considering regional differences. Therefore, this paper developed a meta undesirable two-stage EBM DEA model to analyze the energy, environment, health, and media communication efficiencies in 31 Chinese cities, from which it was found that the productivity efficiency in most cities was better than the health treatment efficiencies, the GDP and fixed asset efficiency improvements were small, the air quality index (AQI) and CO_2_ efficiencies varied widely between the cities, media report and governance inputs were generally inefficient, the birth rate efficiencies were better than the respiratory disease efficiencies, and the technical gap was best in Guangzhou, Shanghai, and Lhasa. Also, it found that high-income cities have a higher technology gap than upper middle–income cities, and media reports efficiency have a high correlation with respiratory diseases and CO_2_.

## 1. Introduction

The World Health Organization [1] claims that air pollution affects 93% of the world’s children’s health and that it is a primary cause of respiratory diseases, especially in low- and middle-income countries. Although heavy industrial development has contributed significantly to Chinese rapid economic growth, it has also resulted in China becoming the world’s largest emitter of greenhouse gases in less than two decades. The American Cancer Society [2] reported that China ranked first in the world in terms of morbidity and mortality from lung, breast, and stomach cancers. As a result of the Chinese government’s “Decision on Strengthening the Cultivation and Development of Strategic Emerging Industries,” the “Air Pollution Prevention and Control Action Plan,” the “Water Pollution Prevention and Control Action Plan,” and the “13th Five-Year Plan Outline” in 2014, environmental pollution control investment reached 975.55 billion CNY. However, in 2015, investment declined and had risen to only 935.39 billion CNY by 2017. From 2006 to 2017, the compound annual growth in China was 12.68% [3]. Generally, national, provincial, and local governments have been using the media to promote environmental pollution reductions and regional health, and therefore, environmental pollution, government governance, health and media communications have become a key focus of discussion.

Past studies have focused on the relationship between economic growth and air pollution [4,5], the childhood and elderly diseases caused by excessive air pollution [6,7,8,9,10], the effects of exposure to air pollutants on human health [11,12,13,14,15,16,17,18,19,20,21,22,23,24], and protection measures against the effects of air pollution [25,26,27,28,29,30,31]. Other studies have examined the effectiveness of media campaigns in raising societal awareness of environmental issues [32,33,34,35,36,37,38,39].

**Figure 1:**
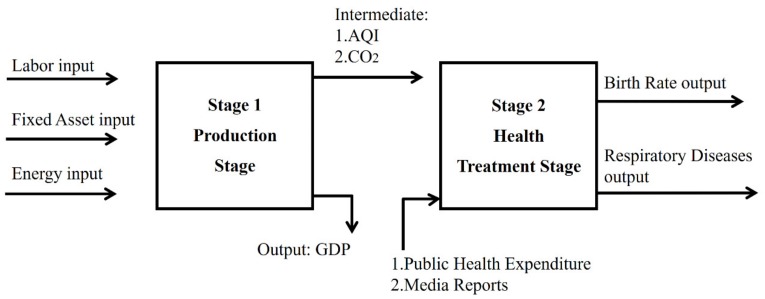
Inputs and outputs in the production and health treatment stage.

However, in general, few studies have linked these energy, air pollution, health discussions and media discourse. This article will therefore discuss economics, environmental pollution, health, and media together. The relationship is shown in Figure 1. In the production stage, labor, energy, and fixed assets create desirable output GDP, but also produce undesirable output CO_2_ and air quality index (AQI) to the environment. The undesirable output CO_2_ and AQI—intermediate output from the production stage (first stage)—can be seen in second stage—input resources—to the production of new values in the health treatment stage (second stage). The government provides health expenditures to reduce diseases caused by environmental pollution. Media reports can objectively present air pollution information, identify the causes of pollution in a timely manner, and identify protective measures so as to prevent or control the risk of physical injury and reduce the incidence of diseases. Media reports can reduce the incidence of public diseases, improve the public’s physical and mental health, and improve the health and ethics of the entire population. So, government health expenditures and media reports can reduce disease. Therefore, energy, environmental pollution, health, and media can be constructed by the two-stage model.

While several past studies have employed network data envelopment analysis (DEA) to examine production and pollution controls [40,41,42,43,44], there has been less research focused on the associations between energy, environmental pollution, health, and the media using a two-stage DEA model. In the past, environmental pollution and energy efficiency analyses have mainly employed radial (such as CCR(named after Charnes, Cooper and Rhodes) or BCC(named after Banker, Charnes, and Cooper)), non-radial (such as SBM (slack-based measures)), or directional distance function (DDFC) DEA models. However, the radial DEA models ignore non-radial slacks, and the non-radial DEA models ignore the same proportion of radial characteristics and do not account for regional differences. Therefore, to fully analyze the effectiveness of government health expenditure inputs and media coverage in combatting the energy and environmental effects of the production/labor first stage inputs, an applied meta two-stage Epsilon-based measure (EBM) DEA model that includes undesirable outputs is developed in this paper to analyze the energy, health, and media reporting efficiencies in 31 mainland Chinese cities.

As this model not only includes existing production efficiencies but also considers the sustainability of human health, it has two main contributions. First, to avoid under- or over-estimation, the economic, environmental, media communication, and health efficiencies are jointly analyzed. Second, a meta undesirable two stage EBM DEA model is used to avoid the radial and non-radial biases. Sometimes, decision making units (DMUs) are not homogeneous and have different resource endowments, such as income and location, which affects the inputs and outputs of DMUs, so the concept of meta should therefore be adopted to avoid large regional difference by grouping DMUs. Data from 2013–2016 for 31 Chinese cities were extracted and analyzed, with production taken as the first stage based on labor, fixed assets, and energy consumption inputs and GDP output, with the link between the production stage and the health treatment stage variables being CO_2_ emissions and AQI. Health treatment was taken as the second stage based on health expenditure and media report inputs and birth rate and respiratory disease prevalence outputs.

## 2. Literature Review and Research Hypotheses

Past research can be divided into discussions on the relationships between economic growth and air pollution and between air pollution and human health, examinations of preventative measures to reduce the effects of air pollution on societal health, and the effect of public awareness raising by the media on air pollution and environmental issues.

With a focus on economic growth and air pollution, Georgiev and Mihaylo [4] found that the inverse U-shaped relationship between economic growth and pollution did not apply to all gases, that most countries were still on growth curve path, and that SO( sulfur dioxide) emissions followed a U-shaped curve. Wang et al. [5] also found that there was a nonlinear relationship between economic growth and carbon emissions. Analyses of the relationships between human health and air pollution have involved an examination of the rise in the prevalence of air pollution diseases in children and the elderly; for example, Ye et al. [6] used generalized linear models (GLMs) to examine the effects of exposure to higher daily maximum temperatures and air pollutant concentrations in Tokyo. Lee et al. [7] uses a generalized additive model (GAM) to explore the effects of multiple air pollutants on the health of the children under 15 years old in Seoul, finding that nitrogen dioxide and ozone were main contributors to childhood asthma. Pino et al. [8] studied 504 four-month-old infants in southeast Santiago, Chile, in 1996 and found that an increase in 10 μg/m^3^ over a 24-h average particulate matter PM2.5 increased the risk for wheezing bronchitis by 5%. In related studies, Chen et al. [9] used GLMs to explore particulate matter and hospitalization for chronic obstructive pulmonary disease (COPD), finding that PM2.5 had significant effects on COPD, and Penard-Morand et al. [10] examined the impact of air pollution on asthma and allergies on 6672 children aged 9–11 years old in 108 randomly schools in France and found that lifetime allergic rhinitis was positively related to an increase in an exposure to SO_2_, PM10, and O_3_. In examinations on the effects of long-term exposure to air pollutants on human health, Loomis et al. [11] found a positive correlation between lung cancer and PM exposure and other air pollution indicators, Oakes et al. [12] reviewed exposure indicators for multiple pollutants, and Fischer et al. [13] studied the relationship between long-term exposure to air pollution and mortality, finding that every 10 μg/m^3^ increase in PM10 and NO_2_ was significantly associated with non-accidental mortality. In other studies, Kelly and Fussel [14] analyzed the health effects of PM and concluded that effective policies had the potential to reduce air pollution, and Pope et al. [15] found that air pollution increased the risk of disease and death, and that estimates of the diseases caused by PM2.5 contamination and pollution reductions depended on concentration–response functions.

Khafaie et al. [16] used research planning, critical assessment methods, and decentralized models to estimate the relationship between air pollution and health and explain the types and sources of air pollution and the common terminologies used in air pollution epidemiological studies. Khafaie et al. [17] conducted a literature review analysis to determine the adverse health effects of short-term and long-term exposure to outdoor air pollution. In the same area, Pannullo et al. [18] proposed a model averaging method to determine the relationships between carbon dioxide concentrations and cardiopulmonary respiratory mortality in Scotland. Xie et al. [19] assessed the impact of PM2.5 on the Chinese economy and found that China’s GDP loss would be approximately 2.00% by 2030 and the PM2.5 pollution medical expenses would be around $25.2 billion, and Yang et al. [20] examined the nonlinear relationships between environmental air pollution exposure and health effects to inform the development of focused epidemiological and national environmental protection policies. Rich [21] reviewed new research in Beijing, Atlanta, London, and Ireland, demonstrating that population health records could improve epidemiological assessments. Newell et al. [22] studied the effects of particulate matter on cardiopulmonary health in low- and middle-income countries, finding that when the PM2.5 increased by 10 μg/m^3^, the cardiovascular mortality increased by 0.47% and the respiratory mortality increased by 0.57%, when PM10 increased by 10μg/m3, cardiovascular mortality increases by 0.27% and respiratory mortality increased by 0.56%, and that short-term exposure to a particulate environment was associated with an increased incidence of cardiopulmonary disease and an increased mortality in low- and middle-income countries. Zigler et al. [23] explored the impact of air quality and human health in the United States and concluded that PM2.5 had a significant impact on health and that increased air quality would lead to a significant drop in associated chronic obstructive pulmonary disease, heart failure, ischemic heart disease, and respiratory infection mortalities. Kinney [24] found that climate and weather had a significant impact on air pollution distribution; at higher ambient temperatures, ozone and PM2.5 emissions increased; and that the PM caused by wildfires was a serious problem.

To reduce the effects of air pollution on societal health, Torretta et al. [25] proposed a modified strategy for the application and reduction of PM air pollution, Schiavon et al. [26] simulated city road traffic NOx (Nitrogen oxide) emissions using a COPERT(EU standard vehicle emissions calculator ) algorithm model, and Schiavon et al. [27] used a standard search from technical literature and then the Atmospheric dispersion modeling 2000 to calculate the annual average concentration of NOx and benzene. Li et al. [28] used advanced production processes to control urban population growth and proposed an emissions trading system to reduce the economic losses caused by the public health effects of air pollution. He and Ou [29] claimed that it was necessary to determine the marginal pollution emissions abatement costs by first quantifying the sulfur dioxide emissions and then estimate the sulfur dioxide emissions using shadow price theory. They found that China’s pollution emissions trading system needed to cover six sectors, including coal mining and coal washing, at the national level. Jose et al. [30] explored the impact of global climate on citizen health, Lua et al. [31] estimated the adverse health effects of air pollution in China from 2001 to 2017, concluding that people with respiratory diseases needed to live in rural areas, where the pollutant concentrations were relatively low.

In a study on the relationship between media promotion and air pollution, Dworkin and Pijwaka [32] explored air quality changes from 1968 to 1978 in Toronto, Canada, and found that mass media had affected public attitudes and public behavior on environmental topics. It found that, with the public’s attention, air pollution is reduced. Mass media has influence on public awareness of environmental issue. Mayer [33] analyzed newspaper article content from the *New York Times*, the *Los Angeles Times*, and the *Washington Post* over 20 years and found that air pollution was generally explained as being related to asthma. However, the newspapers avoid connecting respiratory disease with environmental problems that reduced the government’s control on air pollution due to low public concern. Elliot et al. [34] studied a national real-time syndrome monitoring surveillance system and demonstrated the impact of short-term air pollution on the public and the potential for mass media coverage to increase the volume of healthcare requests. The research investigated healthcare-seeking behavior during two air pollution episodes in England in March and April 2014. The data from national real-time syndromic surveillance systems, which includes general practitioner (GP) consultations, emergency department attendances, telehealth calls, and ambulance dispatch calls, were used, and the patients were divided by four age groups (0–4, 5–14, 15–64, and 65–70 years of age). The age group 15–64, who are the main user of traditional media and social media, had more healthcare-seeking behavior than other groups during air pollution episodes. Therefore, the positive effect of media reporting on healthcare-seeking behavior is shown in this research. Wang [35] investigated the Chinese social media monitoring of air quality and the public responses. The research collected 63 million messages from one of the largest social media server (Sina Weibo) in China. By Pearson’s correlation coefficient tests, the messages related to air pollution had correlation to air pollution level. About 67% of messages in 170 samples were about air pollution. Media has contribution to air pollution insight. Jiang et al. [36] studied the message of air pollution in social media surveys. According to the message data collected from Sina Weibo, the study found that filtered social media information was strongly correlated with the air quality index in Beijing in 2012. Social media message reflects the public awareness of air pollution. Costa and Kahn [37] studied the relation of typhoid death rate and newspaper media reports from 1890 to 1930 in major cities in the US (New York City, Chicago, Baltimore, Boston, Philadelphia, and Washington DC). By negative binomial regression, it was found that news reports tended to be positively correlated with typhoid mortality. The other finding is that bad news draws more public attention. The public reacted more to the increase in death rate than the decrease in death rate. Murukutla [38] examined the online media information about air pollution from 1 January 2014 to 31 October 2015. When 500 media articles were randomly selected from 6435 articles, the research found that a lot of important information was not mention, such as illness, health risk, and the specific institutions that are responsible stakeholders. The research suggested that true sources of and solutions to air pollution reported by the media can trigger government to take policy action. Schwabe [39] studied the most serious fine particulate air pollution in Beijing. It found that intense media report kept public interest and sustain public discussion on this incident, potentially triggered government policy action and policy adjustment.

The theoretical framework for this study is based on previous study results and the following assumptions: public media reports positively impact public awareness of air pollution and its impact on the environment and human health.

Based on the above literature analysis, this paper makes the following research hypotheses:

**H1**: Public media reports have a correlation with CO_2_ and AQI.

**H2**: Public media reports have a correlation with respiratory diseases.

As can be seen from this brief literature review, much of the previous research has only been on a single level using radial and non-radial methods with little consideration for regional differences. Therefore, to overcome these issues, this paper used a meta undesirable two-stage EBM DEA model to explore the energy, environment, health, and media communication efficiencies in 31 mainland Chinese cities. Due to the difference in income between regions, this paper compares the 31 Chinese cities divided into high- and upper middle–income cities. The hypotheses are as follows:

**H3**: In the efficiencies, high-income cities are higher than upper middle–income cities.

**H4**: In the Technology gap, high-income cities are higher than upper middle–income cities.

## 3. Research Method

After Farrell [45] proposed the production frontier, on the assumption of a fixed-scale remuneration, Charnes et al. [46] proposed a CCR (named after Charnes, Cooper and Rhodes) data envelopment analysis model, which was then extended by Banker et al. [47] to a BCC (named after Banker, Charnes, and Cooper), model that could measure technical efficiency (TE) and scale efficiency (SE). However, as the CCR and BCC were radial DEA models that ignored non-radial slacks when evaluating the efficiency value, Tone [48] proposed a slacks-based measure (SBM), which involved non-radial estimation with a single scalar value with the efficiency value being between 0 and 1, that considered both input and output slacks. However, as the SBM DEA ignored the same proportion of radial characteristics when evaluating the efficiency values, Tone and Tsutsui [49] proposed the Epsilon-based measure (EBM) DEA model that included input, output, and non-oriented modes and that overcame the shortcomings of the radial and non-radial DEA models.

In 2007, Färe et al [50] proposed the Network DEA (NDEA) model, which considered sub-production technologies to be sub-decision units (Sub-DMU), with the optimal solution being determined using traditional CCR or BCC models. While the traditional DEA model saw secondary production technology as a “black box,” the NDEA included these secondary production techniques to explore the impact of the input allocations and intermediates on the production process. To analyze the efficiency of each subprocess, Chen and Zhu [51], Hwang and Kao [52], Kao and Hwang [53]. and Kao [54] divided the entire business process into sub-processes and linked the stages with an intermediate output, which allowed for the efficiency of each stage to be calculated separately to determine which sub-process was responsible for the efficiency losses in the system. Tone and Tsutsui [55] then proposed a weighted slacks-based NDEA model in 2009, in which the links between the various DMU departments were used as the basis for the NDEA model analysis, and in which each department was regarded as a sub-DMU, with the optimal solution being determined using the SBM model.

While the EBM DEA resolved the radial and non-radial issues, it failed to deal with the two-stage problem, and while the NDEA model solved the multi-stage problems, it failed to deal with the radial and non-radial problems. As different countries have different social and cultural backgrounds, economic environments, management models, and production efficiencies, and as manufacturers from different countries have different production technologies, if a traditional DEA assumes that all DMUs have the same technical level in the efficiency evaluation, it may be inappropriate to analyze efficiencies using traditional efficiency evaluation models. This paper, therefore, proposes a model based on Tone and Tsutsui [55], O’Donnell et al. [56] and the two stage modified EBM DEA model [49]—a meta undesirable two-stage EBM DEA model.

### 3.1. The Meta Undesirable Two-Stage EBM DEA Model

This study collected data from 31 Chinese cities. In the first stage, labor, fixed assets, and energy consumption were the inputs, and GDP was the output, with the one-stage and two-stage links being CO_2_ emissions and AQI. In the second stage, health expenditure and media reports were the input, with the output being birth rate and respiratory diseases. As respiratory diseases were seen as undesirable output, Tone and Tsutsui’s [49] EBM model was modified to a meta undesirable two-stage EBM DEA model, the description for which is given below.

In a traditional DEA efficiency evaluation, it is usually assumed that all producers have the same level of production technology; however, in reality, most decision-making units have different production technologies because of different geographical locations, national policies, or social and economic conditions. Battese and Rao [57] and Battese et al. [58] proposed a meta-frontier model that compared the technical efficiency of different groups. The meta frontier model proposed by O’Donnell et al. (2008) [56] was found to be able to accurately calculate group and meta technical efficiency values and the MTR.

*n* DMU and K division, *DMU_j_* = (*DMU*_1_,*DMU*_2_,……..,*DMU_k_*,……….,*DMU_n_*), *m* input *X_j_* = (*X_1j_*, *X_2j_*,……..,*X_mj_*), *s* output, *Y_j_* = (*Y_1j_*, *Y_2j_*,…………..*Y_sj_*), DMU efficiency: Due to management, resource, regulatory, and environmental differences, all firms (N) are made up of DMU groups (*N = N_1_ + N_2_ +…+ N_G_*), with X*_ij_* and Y*_rj_* denoting the input (i = 1, 2, …, m) and with the final output being r *(r = 1, 2, …, S*) for unit *j* (*j = 1, 2, …, N*). Under the meta-frontier, DMU k then chooses an optimal final output weight $u_{r}^{g}$ *(r = 1, 2, …, S*) to attain the highest efficiency; therefore, under a non-oriented EBM, the efficiency of DMU k using the meta undesirable two stage EBM DEA can be determined using the following linear programming.

(1) $$\theta^{**}=\underset{0\eta ,\lambda ,s^{-},s^{+g},s^{-b}}{\min}\frac{\sum_{k=1}^{K}W^{k}\left[\theta_{k}-\varepsilon_{xk}\sum_{g=1}^{G}\sum_{i=1}^{m_{k}}\frac{w_{i}^{-k}s_{i}^{-k}}{x_{i0}}\right]}{\sum_{k=1}^{K}W^{k}\{\eta_{k}+\varepsilon_{yk}\sum_{g=1}^{G}\sum_{i=1}^{S_{1k}}\left[\frac{w_{i}^{+S_{1k}}s_{i}^{+gk}}{y_{i0}}+\sum_{i=1}^{S_{2k}}\frac{w_{i}^{-S_{2k}}s_{i}^{-bk}}{y_{i0}}\right]\}}$$

Subject to

(2) $$X_{i0}=\sum_{g=1}^{G}\sum_{j=1}^{n}X_{ijg}\theta_{jg}-S_{i}^{-}\;\left(i=1\ldots m,\;j=1\ldots n;g=1\ldots G\right)$$

(3) $$Y_{i0}=\sum_{g=1}^{G}\sum_{j=1}^{n}Y_{ijg}\eta_{jg}+S_{i}^{+good}\;\left(i=1\ldots s1,\;j=1\ldots n;g=1\ldots G\right)$$

(4) $$Y_{i0}=\sum_{g=1}^{G}\sum_{j=1}^{n}Y_{ijg}\eta_{jg}-S_{i}^{-bad}\;\left(i=1\ldots s2,\;j=1\ldots n;g=1\ldots G\right)$$

(5) $$\overset{G}{\underset{g=1}{\sum }}\overset{n}{\underset{j=1}{\sum }}\lambda_{jg}=1$$

$\lambda \geq 0,\;S^{-}\geq 0,\;S^{+good}\geq 0,\;S^{-bad}\geq 0$, θ≤1, η≥1

Y: DMU output,

X: DMU input,

$S^{-}$: Slack variable,

$S^{+good}$: Surplus variable,

$S^{-bad}$: Surplus variable,

$W^{-}$: Weight of input i, $\sum W_{i}^{-}=1\;\left(\forall_{i}\;W_{i}^{-}\geq 0\right)$

$W^{+}$: Weight of output S, $\sum W_{i}^{+S1}+\sum W_{i}^{-S2}=1\;\left(\forall_{i}\;W_{i}^{+}\geq 0\right)$

$E_{x}$: Set of radial θ and non-radial slacks,

$E_{y}$: Set of radial η and non-radial slacks.

From the above equations, the overall technological efficiency of the cities can be determined under the meta-frontier, and using Equation (1)–(5), the overall technological efficiency of all high-income and upper middle–income cities can be determined under the meta-frontier undesirable two-stage EBM group frontier model

The high- and upper middle–income cities were divided into g decision-making units, each of which was assigned an optimal output weight; therefore, the DMU efficiency under the group frontier was solved using the following equations.

(6) $$\theta^{g*}=\underset{0\eta ,\lambda ,s^{-},s^{+g},s^{-b}}{\min}\frac{\sum_{k=1}^{K}W^{k}\left[\theta_{k}-\varepsilon_{xk}\sum_{i=1}^{m_{k}}\frac{w_{i}^{-k}s_{i}^{-k}}{x_{i0}}\right]}{\sum_{k=1}^{K}W^{k}\{\eta_{k}+\varepsilon_{yk}\sum_{i=1}^{S_{1k}}\left[\frac{w_{i}^{+S_{1k}}s_{i}^{+gk}}{y_{i0}}+\sum_{i=1}^{S_{2k}}\frac{w_{i}^{-S_{2k}}s_{i}^{-bk}}{y_{i0}}\right]\}}$$

Subject to

(7) $$X_{i0}=\sum_{j=1}^{n}X_{ij}\theta_{j}-S_{i}^{-}\;\;\;\;\left(i=1\ldots m;j-1\ldots n\right)$$

(8) $$Y_{i0}=\sum_{j=1}^{n}Y_{ij}\eta_{j}+S_{i}^{+goood}\;\;\;\;\left(i=1\ldots s1;j=1\ldots n\right)$$

(9) $$Y_{i0}=\sum_{j=1}^{n}Y_{ij}\eta_{j}-S_{i}^{-bad}\;\;\;\;\left(i=1\ldots s2;j=1\ldots n\right)$$

(10) $$\overset{n}{\underset{j=1}{\sum }}\lambda_{j}=1\;\;$$

$$\lambda \geq 0,S^{-}\geq 0,\;\;\;\;S^{+good}\geq 0,\;\;\;\;S^{-bad}\geq 0,\;\theta \leq 1,\;\eta \geq 1$$

As the production frontier for the g groups were included in the meta-frontier, the technical efficiency under the meta-frontier needed to be less than the technical efficiency under the group frontier. The ratio of the two frontiers is called the technology gap ratio (TGR):

(11) $$\mathrm{TGR}=\frac{\theta^{**}}{\theta_{}^{*g}}$$

### 3.2. Fixed Assets, Labor, Energy Consumption, GDP, Health Expenditure, Media, Birth Rate, and Respiratory Diseases Efficiencies

The Hu and Wang [59] total-factor energy efficiency index was used to overcome any possible bias in the traditional energy efficiency indicators. There were eight key features in this efficiency study—fixed assets, labor, energy consumption, GDP, health expenditure, media reports, birth rate, and respiratory diseases. In this study, “I” represented area and “t” represented time. The 10 efficiency models are defined in the following expressions:

(12) $$\mathrm{Fixed}\;\mathrm{Assets}\;\mathrm{Efficiency}\;=\;\frac{\mathrm{Target}\;\mathrm{Fixed}\;\mathrm{Assets}\;\mathrm{input}\;\left(i,\;t\right)}{\mathrm{Actual}\;\mathrm{Fixed}\;\mathrm{Assets}\;\mathrm{input}\;\left(i,\;t\right)}$$

(13) $$\mathrm{Labor}\;\mathrm{Efficiency}\;=\;\frac{\mathrm{Target}\;\mathrm{Labor}\;\mathrm{input}\;\left(i,\;t\right)}{\mathrm{Actual}\;\mathrm{Labor}\;\mathrm{input}\;\left(i,\;t\right)}$$

(14) $$\mathrm{Energy}\;\mathrm{Consumption}\;\mathrm{Efficiency}\;=\;\frac{\mathrm{Target}\;\mathrm{Energy}\;\mathrm{input}\;\left(i,\;t\right)}{\mathrm{Actual}\;\mathrm{energy}\;\mathrm{input}\;\left(i,\;t\right)}$$

(15) $$\mathrm{GDP}\;\mathrm{Efficiency}\;=\;\frac{\mathrm{Actual}\;\mathrm{GDP}\;\mathrm{desirable}\;\mathrm{output}\;\left(i,\;t\right)}{\mathrm{Target}\;\mathrm{GDP}\;\mathrm{desirable}\;\mathrm{output}\;\left(i,\;t\right)}$$

(16) $$\mathrm{Health}\;\mathrm{Expenditure}\;\mathrm{Efficiency}\;=\;\frac{\mathrm{Target}\;\mathrm{Health}\;\mathrm{Expenditure}\;\mathrm{input}\;\left(i,\;t\right)}{\mathrm{Actual}\;\mathrm{Health}\;\mathrm{Expenditure}\;\mathrm{input}\;\left(i,\;t\right)}$$

(17) $$\mathrm{Media}\;\mathrm{Efficiency}\;=\;\frac{\mathrm{target}\;\mathrm{media}\;\mathrm{input}\;\left(i,\;t\right)}{\mathrm{actual}\;\mathrm{media}\;\mathrm{input}\;\left(i,\;t\right)}\;\mathrm{Birth}\;\mathrm{Rate}\;\mathrm{Efficiency}\;=\;\frac{\mathrm{Actual}\;\mathrm{Birth}\;\mathrm{Rate}\;\mathrm{desirable}\;\mathrm{output}\;\left(i,\;t\right)}{\mathrm{Target}\;\mathrm{Birth}\;\mathrm{Rate}\;\mathrm{desirable}\;\mathrm{output}\;\left(i,\;t\right)}$$

(18) $$\mathrm{Respiratory}\;\mathrm{Diseases}\;\mathrm{Efficiency}\;=\;\frac{\mathrm{Target}\;\mathrm{Respiratory}\;\mathrm{Diseases}\;\mathrm{Undesirable}\;\mathrm{output}\;\left(i,\;t\right)}{\mathrm{Actual}\;\mathrm{Respiratory}\;\mathrm{Diseases}\;\mathrm{Undesirable}\;\mathrm{output}\;\left(i,\;t\right)}$$

(19) $${\mathrm{CO}}_{2}\;\mathrm{Efficiency}\;=\;\frac{\mathrm{Target}\;\mathrm{CO}{}_{2}\;\mathrm{Undesirable}\;\mathrm{output}\;\left(i,\;t\right)}{\mathrm{Actual}\;\mathrm{CO}{}_{2}\;\mathrm{Undesirable}\;\mathrm{output}\;\left(i,\;t\right)}$$

(20) $$\mathrm{AQI}\;\mathrm{Efficiency}\;=\;\frac{\mathrm{Target}\;\mathrm{AQI}\;\mathrm{Undesirable}\;\mathrm{output}\;\left(i,\;t\right)}{\mathrm{Actual}\;\mathrm{AQI}\;\mathrm{Undesirable}\;\mathrm{output}\;\left(i,\;t\right)}$$

If the target fixed assets, labor, energy consumption, and health expenditure inputs equaled the actual inputs, then the fixed assets, labor, energy consumption, health expenditure, and media efficiencies equaled 1, indicating overall efficiency. If the target fixed assets, labor, energy consumption, health expenditure, and media inputs were less than the actual inputs, then the fixed assets, labor, energy consumption, health expenditure, and media efficiencies were less than 1, indicating overall inefficiency.

If the target GDP and birth rate desirable outputs were equal to the actual GDP and birth rate desirable outputs, then the GDP and birth rate efficiencies equaled 1, indicating overall efficiency. If the actual GDP and birth rate desirable outputs were less than the target GDP and birth rate desirable outputs, then the GDP and birth rate efficiencies were less than 1, indicating overall inefficiency.

If the target respiratory diseases, CO_2_, and AQI undesirable outputs were equal to the actual respiratory disease, CO_2_, and AQI undesirable outputs, then the respiratory diseases, CO_2_ and AQI efficiencies equaled 1, indicating efficiency. If the target respiratory disease, CO_2_, and AQI undesirable outputs were less than the actual respiratory disease, CO_2_, and AQI undesirable outputs, then respiratory diseases, CO_2_, and AQI efficiencies were less than 1, indicating inefficiency.

## 4. Empirical Study

### 4.1. Data Sources and Description

#### Data and Variables

Based on the World Bank’s classification for rich and poor countries, the 31 Chinese cities were divided into high- and upper middle–income cities, with the upper middle–income economies having a GNI (Gross National Income) per capita between $3896 and $12,055, and the high-income economies having a GNI per capita of $12,056 or more [60].

Therefore, the 31 sample cities were divided into 14 high-income cities (Beijing, Changsha, Fuzhou, Guangzhou, Hangzhou, Huhehot, Jinan, Nanchang, Nanjing, Shanghai, Shenyang, Tianjin, Wuhan, and Zhengzhou) and 17 upper middle–income cities (Chengdu, Changchun, Chongqing, Guiyang, Harbin, Haikou, Hefei, Kunming, Lanzhou, Lhasa, Nanning, Shijiazhuang, Taiyuan, Urumqi, Xian, Xining, and Yinchuan).

**Figure 2:**
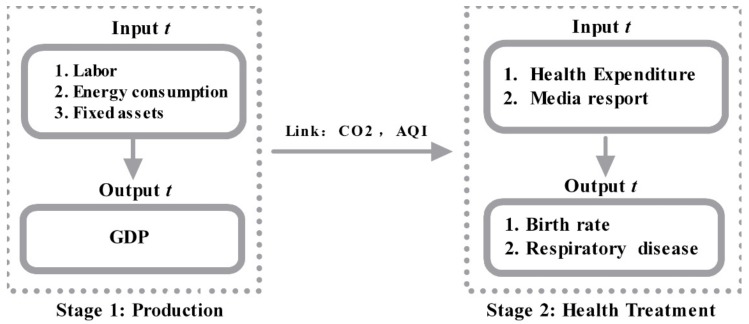
Network data envelopment analysis (DEA) index.

Data from 2013 to 2016 were extracted from the Statistical Yearbooks of China [61], the Demographics and Employment Statistical Yearbooks of China [62], and the Statistical yearbooks from each city. Air pollutant data were collected from China Environmental Protection Bureau reports [3]. The research follows past research on energy and environment, the inputs are labor, fixed assets, and energy consumption (Hu and Wang [59], Wang and Wei [63], Du et al. [64], Li et al. [65]). GDP is desirable output, and CO_2_ and AQI are undesirable outputs (Yeh et al. [66], Choi et al. [67], Wang et al. [68], Wang and Wei [63]). In the second stage, following Zhang et al. [69], input is health expenditure and output is birth rate. The link variables of two stages are CO_2_ and AQI, which also followed Zhang et al. [69]. The two-stage model is shown in Figure 2

The variables used in the study are explained in the following:

First stage: production stage

Input variables:

- Labor input: Employees; this study used the number of employed people in each city at the end of each year; unit: person.
- Energy consumption was calculated from the total energy consumption in each city; unit: 100 million Tonnes.
- Fixed assets: the capital stock in each city was calculated using the fixed assets investment in each city; unit: 100 million CNY.

Output variables:

Desirable output (GDP): the GDP in each city was used as each city’s output; unit: 100 million CNY;

Link Production Stage and health stage variables:

- Carbon dioxide: CO_2_, a common greenhouse gas;
- AQI: the air quality index, which is a measured concentration of the pollutants such as PM2.5, PM10, sulfur dioxide (SO_2_), and nitrogen.

Second stage: health treatment stage

Input variables:

- Health expenditure: the total amount of health care invested in each place. Since this study is unable to obtain medical input for different diseases, the study can only use the total amount of medical input and conduct research based on the efficiency changes in each region;
- Media reports: media reports can reduce the incidence of public diseases and improve the public’s physical and mental health. The media reports collected in this study are from the People’s Daily Online, Xinhuanet, and Sina Weibo of the People’s Daily, Beijing News, Caixin.com, China Youth Daily, and China News Weekly. These media are official Chinese state-owned media, and the reliability of the report is strong. Related air pollution news data were collected from the Xinhuanet media official website using the search string “province + air pollution.” Statistics were calculated in units of (years), with the number of statistics being the total amount in the year. The official news websites were selected because of the amount of news published and their wide influence.

Output variables:

- Respiratory Diseases: referring to the prevalence of respiratory diseases. In order to examine the effects of medical health inputs on diseases in various regions, only respiratory disease data can be collected, and data on specific respiratory diseases cannot be obtained. Therefore, in this study, the respiratory disease rate was used to measure the adverse effects of air pollutants and the effects of health management, mainly because a large amount of literature research has been proved. Some researchers had found that respiratory disease is significantly affected by air pollutants as PM10 (small dust particles). In some model specifications, ozone, another measure of pollution, is also found to affect respiratory illness. Furong et al. [70] studied data from 2009 to 2015 in Hefei, China, and showed that air pollution can significantly increase the mortality of respiratory diseases and lung cancer. Among them, the mortality rate of lung cancer is significantly correlated with SO_2_. Karimi et al. [71] collected the data of mortality and hospitalization rates for cardiovascular and respiratory diseases associated with air pollution from January 1980 to January 2018 in the PubMed, EMBASE, and Web of Science databases. The research used systematic review and meta-analysis to explore the relationship between air pollution, cardiovascular, respiratory mortality, and hospitalization rates. The results showed that air pollutants (O_3_, PM2.5, PM10, NO_2_, NOx, SO_2_, and CO) were associated with increased mortality and hospitalization rates, but PM2.5 and PM10 were more strongly affected.
- Birth rate: this study used the infant birth rate as the second output indicator for medical input. Carré [72] explored that air pollution, especially PM2.5, PM10, and NO_2_, had significant effects on female fertility and infant birth rate.

### 4.2. Basic Statistical Analysis

**Figure 3:**
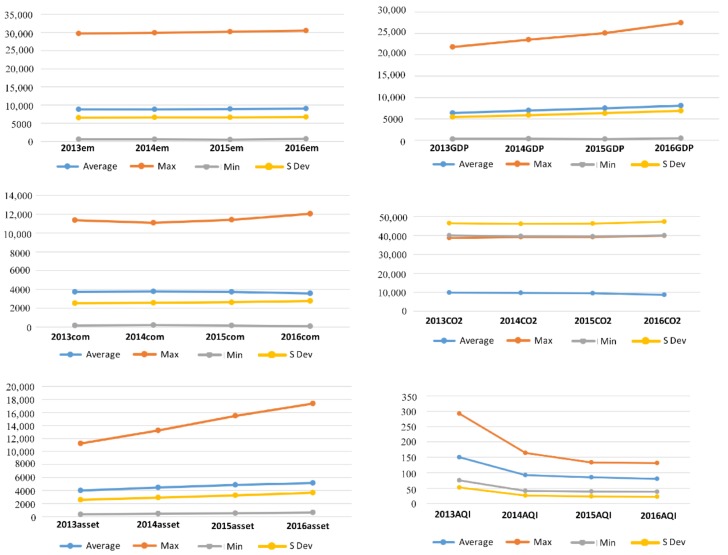
Statistical analysis of labor, fixed assets, energy consumption, AQI, CO_2_, and GDP.

Figure 3 shows the input and output statistics in the first production stage. The most significant increases were in GDP and fixed assets, with the increase in the maximum fixed assets value being particularly significant, reflecting the fact that economic growth has relied mainly on sustained fixed assets investment in the past few years. The difference between the maximum and minimum values was increasing. Labor grew slowly, with the difference between the maximum and minimum increasing. Energy consumption fluctuated and declined in 2014, but the maximum rose to new heights from 2015 to 2016, with the average energy consumption declining from 2015 to 2016.

The average value of the undesirable carbon dioxide emissions fell in 2016, the maximum dropped in 2015, and then rose again in 2016. The maximum and average AQI reached its highest point in 2013 and then continuously declined. The minimum value decreased in 2016 but was slightly smaller compared with the maximum.

**Figure 4:**
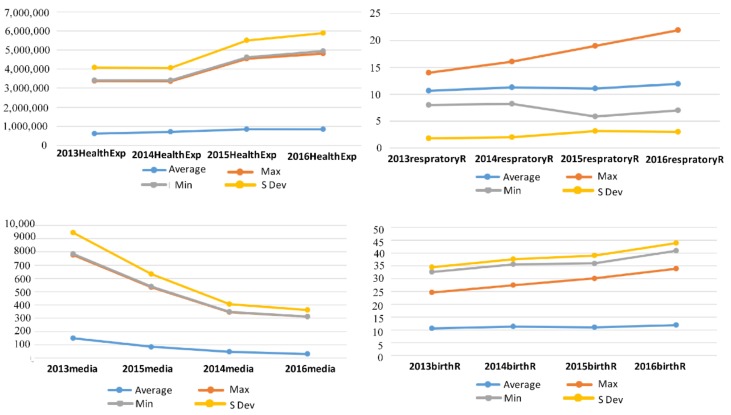
Health expenditure, media reports, respiratory diseases, and birth rate statistics.

Figure 4 shows the second stage health expenditure, media report, respiratory disease, and birth rate efficiencies. Health expenditure had a significant increase after 2014, and although the average remained stable from 2015 to 2016, the maximum and standard deviations increased significantly.

The maximum respiratory disease efficiency continued to rise, and the average value decreased in 2015 and increased slightly in 2016. The birth rate efficiency was the highest, with both the minimum and average showing a continuous slow rise. The standard deviation reached a high in 2015 and declined slightly in 2016.

After reaching a peak in 2013, the maximum, minimum, and average news report efficiencies continued to decline and dropped significantly to the lowest point in 2014. From 2013, when mainland China began to disclose the annual AQI data in each region, the media attention on air pollution began to decline, and the reports decreased.

### 4.3. Overall Efficiency Analysis

**Figure 5:**
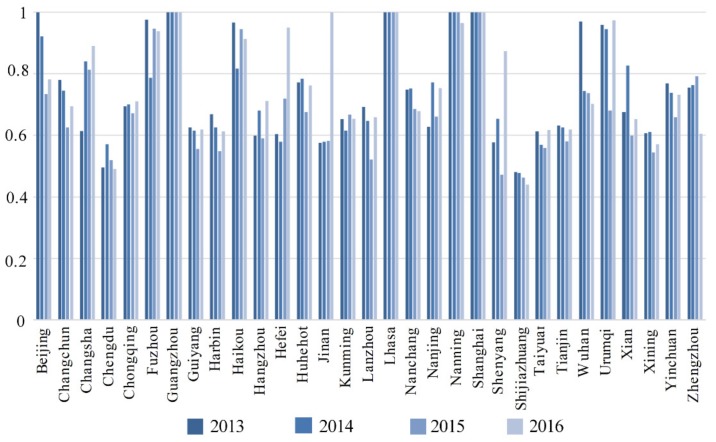
Overall efficiencies in the 31 Chinese cities from 2013 to 2016.

In Table 1 and Figure 5, as can be seen, there were significant efficiency differences across the cities. The best overall efficiencies over the four years were Guangzhou, Lhasa, and Shanghai. Beijing’s overall efficiency only attained 1 in 2013, and there were significant improvements needed in the other cities.

Chengdu and Shijiazhuang had efficiency fluctuations, with Chengdu’s fluctuating below 0.6 for all four years, and with Shijiazhuang’s being below 0.5 and having a downward trend.

Five cities had efficiencies above 0.8 for three years, and four cities had efficiencies between 0.5 and 0.8 for four years. Nanning’s efficiency was 1 in three years, which fell slightly in 2016 to around 0.9. Beijing achieved an efficiency of 1 in 2013, after which it began to decline, reaching its lowest value in 2015 at 0.8.

### 4.4. Efficiency Analysis of the Production and Health Treatment Stages

Table 2 shows the efficiencies for the cities in the production and health treatment/media impact stages from 2013 to 2016.

In the production stage, there were annual efficiencies of 1 only in Guangzhou, Lhasa, and Shanghai in all four years, and in the treatment stage, there were annual efficiencies of 1 in Fuzhou, Guangzhou, Haikou, Lhasa, Nanning, and Shanghai.

Beijing, Changchun, Changsha, Chengdu, Harbin, Hangzhou, Hefei, Hohhot, Jinan, Nanchang, Nanjing, Shenyang, Tianjin, Wuhan, and Zhengzhou had higher efficiency scores in the production stage than the treatment stage, and Chongqing, Guiyang, Haikou, Kunming, Lanzhou, Nanning, Shijiazhuang, Taiyuan, Urumqi, Xi’an, Xining, and Yinchuan had higher efficiency scores in the treatment stage than the production stage.

Chongqing, Guiyang, Kunming, Lanzhou, Shijiazhuang, Taiyuan, Xining, and Yinchuan had four-year production stage efficiencies below 0.6, with the poorest being in Shijiazhuang, with four-year efficiencies of around 0.4. All other cities had efficiencies between 0.4 and 0.6.

The worst performing cities in the treatment stage were Chengdu and Tianjin, with Chengdu having an efficiency of less than 0.4 in three years; therefore, there was a significant need for improvement.

The declines in the production stage were much smaller than in the treatment stage, with the largest being in Nanchang, which fell from 0.85 in 2013 to 0.66 in 2016. Efficiency changes in the treatment stage were more volatile, with Beijing and Wuhan having the largest declines, from 1 in 2013 to 0.6 in 2016.

The efficiency increases in both the production and treatment stages were much the same. Changsha, Hefei, Jinan had the largest increases, with Jinan rising from 0.47 in 2013 to 1 in 2016 and Hefei rising from 0.48 in 2013 to 1 in 2016.

In Table 3, the Wilcoxon Test shows that the total efficiency of high-income and upper middle–income countries from 2013 to 2016 is weak significant. The total efficiency of 2014 is not significant, but the total efficiency of 2014 to 2016 is weakly significant, which is consistent with the H3 hypothesis.

In production stage, according to the Wilcoxon Test, the efficiency of the high-income and upper middle–income countries from 2013 and 2015 is strong significant. The efficiency of the 2014 and 2016 production stages is not significant, but the efficiency of the 2013 and 2015 production stages is strongly significant. The efficiency values of high-income countries are higher than those of upper middle–income countries, consistent with the H3 hypothesis in 2013 and 2015.

In treatment stage, Wilcoxon Test shows that the efficiency of high-income and upper middle–income countries from 2013 and 2015 is strongly significant. The efficiency of the treatment stage in 2014 and 2016 is not significant, but the efficiency of the treatment stage in 2013 and 2015 is strongly significant. The efficiency values of high-income countries are higher than those of upper middle–income countries, consistant with the H3 hypothesis in 2013 and 2015.

### 4.5. Efficiency Analysis of the Indicators in the 31 Cities from 2013 to 2016

Table 4 shows the labor, fixed assets, and energy consumption efficiencies, from which it can be seen that the worst performances were in fixed assets, followed by energy consumption, and labor efficiency, which was relatively good.

Only Guangzhou, Lhasa, and Shanghai had fixed assets efficiencies of 1 in all four years; however, Beijing, Haikou, Nanning, and Urumqi all had annual efficiencies higher than 0.8. The other 24 cities had a significant need for improvement. For example, Changsha, Chongqing, Guiyang, Hefei, Kunming, Nanchang, Nanning, Shijiazhuang, Tianjin, Xi’an, and Yinchuan all had efficiencies under or around 0.6, with Tianjin requiring the most improvements at only 0.45 in 2013.

Guangzhou, Lhasa, Nanning, and Shanghai had energy consumption efficiencies of 1 in all four years, and Beijing, Changchun, Fuzhou, Harbin, Haikou, Hefei, Nanchang, Urumqi, and Zhengzhou all had efficiencies higher than 0.8. However, Guiyang, Lanzhou, Shijiazhuang, Taiyuan, Xining, and Yinchuan had efficiencies lower that 0.6, with the worst performance being in Taiyuan, at below 0.2, followed by Shijiazhuang, Lanzhou, and Yinchuan at around 0.4.

Only Guangzhou, Lhasa, and Shanghai had labor efficiencies of 1 for all four years, and the worst performing cities were Chongqing, Guiyang, Kunming, Lanzhou, Shijiazhuang, Taiyuan, and Xining at below 0.7 in most years. All other cities had labor efficiencies between 0.8 and 0.9.

Table 5 shows the GDP, carbon dioxide emissions, and AOI efficiencies in each city, from which it can be seen that there were large differences.

Fuzhou, Guangzhou, Haikou, Lhasa, Nanning, and Shanghai had carbon dioxide emissions efficiencies of 1 in all four years. However, Lanzhou, Taiyuan, and Yinchuan all scored less than 0.4, with all of Taiyuan’s results below 0.2. Changchun, Harbin, Hefei, Nanchang, Urumqi, and Zhengzhou had carbon dioxide emissions efficiencies higher than 0.8, and the other cities had carbon dioxide emissions efficiencies between 0.6 and 0.8.

There were also large differences in the AQI efficiencies. Beijing. Chongqing, Fuzhou, Guangzhou, Haikou, Kunming, Lhasa, Nanjing, Nanning, Shanghai, and Urumqi had AQI efficiencies of 1 in all four years, and many cities had two- or three-year efficiencies of 1, with the other years being above 0.9. However, the AQI efficiencies in Lanzhou, Taiyuan, Xining, and Yinchuan began to decline from 2013 and, by 2016, had fallen to around 0.4. The largest declines were in Lanzhou, Nanchang, Shijiazhuang, Taiyuan, Wuhan, Xi’an, Xining, Yinchuan, and Zhengzhou, but there were AQI efficiency increases in Changchun, Chengdu, Harbin, Hefei, Jinan, Urumqi, and Shenyang.

The GDP efficiencies were better than the CO_2_ emissions and AQI efficiencies in most cities. Guangzhou, Lhasa, and Shanghai had GDP efficiencies of 1, and the GDP efficiencies in Beijing and Nanning in the first three years were all 1, but both declined slightly in 2016. Guiyang, Kunming, Lanzhou, Shijiazhuang, Taiyuan, and Xining had comparatively poor efficiencies at lower than 0.8, and all other cities had GDP efficiencies between 0.8 and 1.

Table 6 shows the health expenditure, media report, respiratory diseases, and birth rate efficiencies in the treatment stage. Fuzhou, Guangzhou, Haikou, Lhasa, Nanning, and Shanghai had media report efficiencies of 1 in all four years. Changchun, Guiyang, Hohhot, Kunming, Wuhan, Urumqi, and Xi’an had media report efficiencies higher than 0.8 in three years. Changsha, Chengdu, Harbin, Hangzhou, Hefei, Nanchang, Nanjing, Shenyang, and Tianjin had media report efficiencies between 0.5 and 0.7. The worst performances were in Lanzhou and Xining, with media report efficiencies of only 0.4 per year, and Shijiazhuang, Yinchuan, and Zhengzhou had media report efficiencies only slightly higher than 0.4 in one or two years. The efficiencies in 10 cities had volatile declines, and the media report efficiencies in the other 13 cities fluctuated up.

Fuzhou, Guangzhou, Haikou, Lhasa, Nanning, Shanghai. Beijing, Changsha, Urumqi, Xining, and Yinchuan had health expenditure efficiencies of 1 or at least two years above 0.9. However, Zhengzhou’s health expenditure efficiency in all four years was below 0.5, and Tianjin had a health expenditure efficiency less than 0.2 for three years. There were noticeable health expenditure efficiency volatilities. However, 13 cities had reduced efficiencies and needed improvements.

Fuzhou, Guangzhou, Haikou, Lhasa, Nanjing, and Shanghai had birth rate and respiratory diseases efficiencies of 1, but Chengdu, Harbin, Shijiazhuang, and Tianjin had four-year efficiencies just above 0.7.

Compared with the birth rate efficiency, the respiratory diseases efficiencies required significant improvements. Fuzhou, Guangzhou, Haikou, Lhasa, Nanning, and Shanghai had respiratory disease efficiencies of 1, Chengdu and Tianjin had respiratory disease efficiencies of around 0.6, and Harbin and Shenyang had three-year efficiencies between 0.7 and 0.8. Nine cities had reduced efficiencies, and 17 cities had rising efficiencies.

According to Table 7, in 2013, the correlation coefficient between media efficiency and CO_2_ and AQI efficiency exceeded 0.4 (at significant level p-value less than 0.05), and there is a high correlation, which is consistent with the H1 hypothesis. The correlation coefficient between Media efficiency and Respiratory Diseases efficiency is 0.5932 (at significant level *p*-value less than 0.05), which is correlated and conforms to the H2 hypothesis.

In 2014, the correlation coefficient between media efficiency and CO_2_ and AQI efficiency are 0.4275 and 0.3387 (at significant level *p*-value less than 0.1), and there is a high correlation, which is consistent with the H1 hypothesis. The correlation coefficient between Media efficiency and respiratory diseases efficiency is 0.4252 (at significant level *p*-value of less than 0.05), which is correlated to and conforms to the H2 hypothesis.

In 2015, the correlation coefficient between media efficiency and CO_2_ and AQI efficiency exceeded 0.4 (at significant level *p*-value of less than 0.05), and there is a high correlation, which is consistent with the H1 hypothesis. The correlation coefficient between media efficiency and respiratory diseases efficiency is 0.5751 (at significant level p-value less than 0.05), which is correlated to and conforms to the H2 hypothesis.

In 2016, the correlation coefficient between media efficiency and CO_2_ and AQI efficiency exceeded 0.4 (at significant level *p*-value of less than 0.05), and there is a high correlation, which is consistent with the H1 hypothesis. The correlation coefficient between media efficiency and respiratory diseases efficiency is 0.3697 (at significant level *p*-value of less than 0.05), which is correlated and conforms to the H2 hypothesis.

### 4.6. Technology Gap Ratio and the Two-Stage Technology Gap Ratio in Each City

**Figure 6:**
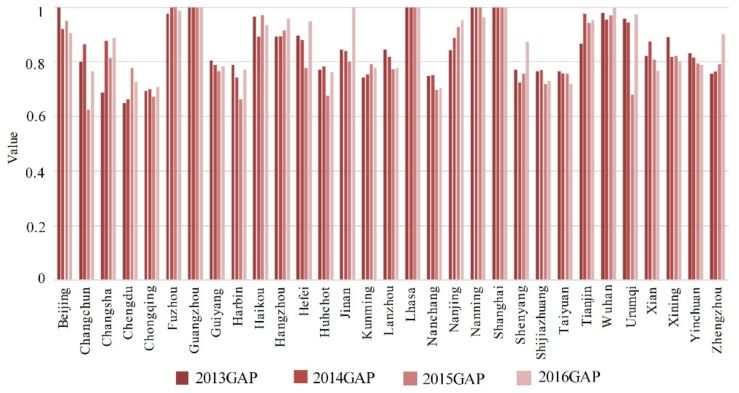
Technology gap ratio in cities from 2013 to 2016.

Table 8 and Figure 6 show the technological frontier in the production and treatment stages from 2013 to 2016. The technology frontier was 1 in Guangzhou, Lhasa, and Shanghai, and there were large differences in the other cities.

Chengdu, Chongqing, Nanchang, Shijiazhuang, and Taiyuan had technology frontiers of 0.7, which fell in Nanchang, Shijiazhuang, and Taiyuan. However, the technology frontier in the other cities was mostly between 0.8 and 0.9.

Changsha, Chengdu, Chongqing, Fuzhou, Hangzhou, Hefei, Jinan, Kunming, Nanjing, Shenyang, Wuhan, Urumqi, and Zhengzhou had rising technology frontiers, but they fell in the other 15 cities, indicating that the technology gap between the cities was expanding, which is in line with the economic development and technical level characteristics in mainland China.

#### Technology Gap between the Production Stage and the Health Management Stage in Each City

Table 9 shows the production and treatment stage technology gaps in each city from 2013 to 2016, from which it can be seen that Guangzhou, Lhasa, and Shanghai had technology frontiers of 1 in the production stage, and Beijing, Fuzhou, Hangzhou, Nanjing, Nanning, Tianjin, Wuhan, and Zhengzhou were close to 1. However, there were large technology gap differences in Chengdu, Chongqing, Guiyang, Kunming, Lanzhou, Shijiazhuang, Taiyuan, and Yinchuan. While these cities were leading in their own regions, their technology frontier required significant improvements to catch up with other cities.

Fuzhou, Hefei, Jinan, Kunming, Shenyang, Wuhan, Urumqi, and Zhengzhou had rising technology frontiers during the production stage, but those of the other cities fell, further indicating that the technology gap between the regions was expanding. For example, while Harbin, Hohhot, Jinan, Nanchang, Shenyang, and Zhengzhou had higher technology gaps compared to cities in their regions, there was still a large gap compared with other cities in the country. Only Beijing and Shijiazhuang had declining technology gaps; however, the scores in the other 23 cities all rose, indicating that the technology gap between the regional cities in the treatment stage in most cities shrank.

According to the Wilcoxon Test in Table 10, high-income and upper middle–income countries Total technology gap is strong significant from 2013 to 2016. In 2014 Total technology gap is week significant. The technology gap of high-income countries is higher than that of upper middle–income countries, consistent with the H4 hypothesis.

In production stage, Wilcoxon Test shows that the technology gap of strong-income and upper middle–income countries from 2013 to 2016 is strongly significant. In other word, the technology gap of high-income countries is higher than that of upper middle–income countries, consists with the H4 hypothesis.

In the treatment stage, the Wilcoxon Test shows that the technology gap of high-income and upper middle–income countries is strongly significant from 2013 to 2016, where the technology gap of the 2014 treatment stage is weakly significant. The treatment stages in 2013, 2015, and 2016, technology gap is strongly significant. The technology gap of high-income countries is higher than that of upper middle–income countries, consistent with the H4 hypothesis.

## 5. Conclusions and Policy Recommendations

This study used panel data and a meta undesirable two-stage EBM DEA to examine the production and health governance efficiencies in 31 Chinese provincial capital cities from 2013 to 2016 under the influence of media reports and the technology gap between high-income and middle-income cities.

The main conclusions from this study were as follows:

1. Guangzhou, Lhasa, and Shanghai had overall efficiencies of 1. Beijing’s overall efficiency score was only 1 in 2013 but was lower in other years, and the other 20 cities had four-year efficiency scores between 0.5 and 0.8; therefore, most cities needed efficiency improvements.
2. Guangzhou, Lhasa, and Shanghai had annual efficiencies of 1 in the production stage, and Fuzhou, Guangzhou, Haikou, Lhasa Nanning, and Shanghai had annual efficiencies of 1 in the treatment stage. Overall, 15 cities had higher efficiencies in the production stage than in the treatment stage, and 12 cities had higher efficiencies in the treatment stage than in the production stage. Chongqing, Guiyang, Kunming, Lanzhou, Shijiazhuang, Taiyuan, Xining, and Yinchuan had four-year production stage efficiencies below 0.6, with the poorest being Shijiazhuang, with a four-year efficiency of around 0.4. Chengdu and Tianjin had the poorest treatment efficiencies; however, in general, the treatment stage and production stage efficiencies were similar.
3. Guangzhou, Lhasa and Shanghai had fixed assets efficiencies of 1 in all four years, but 11 cities had four-year fixed assets efficiencies of only about 0.6, with Tianjin, which had a four-year efficiency of below 0.45, requiring the most improvement. Guangzhou, Lhasa, Nanning, and Shanghai had energy consumption efficiencies of 1 in all four years, nine cities had efficiencies higher than 0.8, and the lowest efficiency was in Taiyuan, at below 0.2. Guangzhou, Lhasa, and Shanghai had labor efficiencies of 1, and only seven other cities had labor efficiencies below 0.7 in most years.
4. Fuzhou, Guangzhou, Haikou, Lhasa, Nanning, and Shanghai had carbon dioxide emissions efficiencies of 1 in all four production stage years, but the other 25 cities had carbon dioxide emissions efficiencies of less than 0.4 in all four years, of which Taiyuan had the lowest, at less than 0.2. Only Beijing had an AQI efficiency of 1 in all four years, and Chongqing, Fuzhou, Guangzhou, Haikou, Kunming, Lhasa, Nanjing, Nanning, Shanghai, and Urumqi had two- or three-year efficiencies of 1, with the other years being higher than 0.9. However, in most cities, the AQI efficiencies had large fluctuations, with eight cities fluctuating upward. Even though only three cities achieved GDP efficiencies, the efficiencies were relatively good in most cities, at close to 0.8.
5. Fuzhou, Guangzhou, Haikou, Lhasa, Nanning, and Shanghai had media report efficiencies of 1 in all four years, and the efficiencies in 17 cities ranged from 0.5 to 0.9 in most years. However, Lanzhou and Xining’s highest annual efficiencies were only about 0.4. The media report efficiencies fluctuated significantly, and many cities experienced large declines, with the largest being in Beijing.
6. Fuzhou, Guangzhou, Haikou, Lhasa, Nanning, and Shanghai had four-year health expenditure efficiencies of 1. However, Tianjin had the worst performance, with its health expenditure efficiency in three-years being below 0.2. The health expenditure efficiencies in the other cities fluctuated significantly, and many cities experienced large declines, with the largest being in Beijing. The urban birth rate efficiency improvements were small; however, Chengdu, Harbin, Shijiazhuang, and Tianjin had the lowest four-year efficiencies at above 0.7.
7. The respiratory disease efficiencies required in most cities needed significant improvements, and the efficiency differences between the cities was wide. Fuzhou, Guangzhou, Haikou, Lhasa, Nanning, and Shanghai had four-year respiratory disease efficiencies of 1. Chengdu and Tianjin had low efficiencies of around 0.6 in most years. Nine cities had four-year efficiency fluctuations or declines, and the other 17 cities had upward fluctuations or continuous upward trends. Overall, the respiratory disease efficiencies improved.
8. The media reports efficiency has a high correlation with **respiratory** diseases, AQI, and CO_2_ efficiency.
9. Guangzhou, Lhasa, and Shanghai had technology frontiers of 1, but Chengdu, Chongqing, Nanchang, Shijiazhuang, and Taiyuan had large technology gaps. Changsha, Chengdu, Chongqing, Fuzhou, Hangzhou, Hefei, Jinan, Kunming, Nanjing, Shenyang, Wuhan, Urumqi, and Zhengzhou had rising technology frontiers, but the technology frontiers in the other 15 cities fell.
10. Fuzhou, Guangzhou, Guiyang, Haikou, Lhasa, Nanjing, and Shanghai had treatment stage technology frontiers of 1. However, Harbin, Hohhot, Jinan, Nanchang, Shenyang, and Zhengzhou had backward technology frontiers. Beijing and Shijiazhuang had sustained fluctuating technology frontiers, and the technology frontiers in the other 23 cities all rose, indicating that the technological differences in the treatment stage shrank. Also, we found that high-income cities are higher technology gap than upper middle–income cities.

From these results, the following policy recommendations are given.

1. As there were obvious differences between the cities, cooperation between regions should be actively promoted. The technology gap of high-income countries is higher than that of upper middle–income countries. So, high-income cities have technological advantages and rich experience in air pollution and health management. High-income cities can use advanced technologies for air pollution treatment by combining regional characteristics, economic and social development levels, geographical characteristics of cities, and meteorological conditions.
2. Industrial structure and energy structure adjustments need to be more rapidly implemented to improve the production and environmental efficiencies in the Beijing-Tianjin region. The Beijing-centered Beijing-Tianjin region had lower production and treatment stage efficiencies than the Pearl River Delta area with Guangzhou at the center and the Yangtze River Delta area with Shanghai at the center. Therefore, the energy consumption, fixed assets investment, human resource input, and environmental efficiencies need to be improved in Beijing. The economic and energy structures in the Beijing-Tianjin region are closely related, and the economic growth in the region has relied heavily on coal for its energy production. Therefore, there needs to be a greater focus on energy structure adjustments and clean energy and clean coal–use technological developments to replace coal and maintain production. Developing and maintaining normal economic and social development is also an important treatment measure.
3. The media is an important “link” and “bridge” for the dissemination of health information. It plays an irreplaceable role in reporting health knowledge, changing health concepts, and promoting healthy behavior. The media has strengthened coverage of air pollution, energy conservation and emission reduction, green development, and environmental protection in terms of content and channels. By continuously improving the scientific and professional reporting of the media, this can guide the public to rationally think about and interpret information and improve the accumulation of public health knowledge. On the other hand, it eliminates public fears and threats of air pollution and promotes public health awareness. Therefore, it is important to enhance the strategy of media coverage. In order to increase the effect of media reporting on public health, media organizations need to constantly improve and strengthen reporting strategies. The media needs to strengthen reports on air pollution, energy conservation, green development, and environmental protection in terms of content, channels, and forms of communication and needs to improve the science and professionalism of the reporting that guides the public to think about and interpret information rationally.
4. Drawing on the advanced health management efficiency in the Pearl River Delta and the Yangtze River Delta, the government can enhance the health management efficiency in the Beijing-Tianjin region. Health management investment in the Beijing-Tianjin region needs to continue to increase in line with economic growth and social development. The Beijing-Tianjin region still needs to strengthen its overall management, improve governance, and design more effective systems to improve health management efficiency.
5. The governance in the middle-income cities needs to adopt strategies and measures appropriate to the regional characteristics. Middle-income cities in the west, such as Lanzhou, Xining, and Yinchuan, need to strengthen their industrial and energy structure adjustments. The news reporting efficiencies in these cities also need significant improvement. Therefore, systems need to be developed that are more suitable to the energy, economic, social, environmental and news reporting characteristics in these cities.
6. To improve their production efficiency and environmental efficiencies, middle-income cities in the midwest and some individual middle-income cities in the East (Changsha, Chengdu, Chongqing, Fuzhou, Hangzhou, Hefei, Jinan, Kunming, Nanjing, Shenyang, Wuhan, Urumqi, and Zhengzhou) need to learn from the advanced technologies in cities such as Guangzhou and Shanghai.
7. Middle-income cities in the northeast and some central cities (Harbin, Hohhot, Jinan, Nanchang, Shenyang, and Zhengzhou) need to improve their news report and health governance efficiencies. Therefore, these cities could learn from the governance measures adopted in other cities to improve the effectiveness of their news reports to increase the environmental awareness of their residents.

## Figures and Tables

**Table 1:** Overall efficiencies in the 31 Chinese capital cities from 2013 to 2016.

NO	DMU	2013	2014	2015	2016
1	Beijing	1	0.920417	0.733929	0.781173
2	Changchun	0.780636	0.744334	0.626083	0.693914
3	Changsha	0.613127	0.83919	0.813482	0.888706
4	Chengdu	0.495873	0.569804	0.518157	0.491098
5	Chongqing	0.692683	0.700274	0.67162	0.709513
6	Fuzhou	0.976286	0.786121	0.945944	0.93785
7	Guangzhou	1	1	1	1
8	Guiyang	0.625708	0.615078	0.556014	0.619227
9	Harbin	0.667707	0.625093	0.548797	0.612453
10	Haikou	0.965631	0.816178	0.944715	0.912714
11	Hangzhou	0.599453	0.680341	0.589847	0.711524
12	Hefei	0.603771	0.578212	0.718987	0.948708
13	Huhehot	0.771201	0.783619	0.675695	0.761003
14	Jinan	0.576035	0.578343	0.582106	1
15	Kunming	0.651187	0.616113	0.667184	0.652977
16	Lanzhou	0.691725	0.647571	0.520495	0.657953
17	Lhasa	1	1	1	1
18	Nanchang	0.747725	0.751731	0.684811	0.678634
19	Nanjing	0.626384	0.771059	0.660945	0.752753
20	Nanning	1	1	1	0.963501
21	Shanghai	1	1	1	1
22	Shenyang	0.57641	0.653371	0.471707	0.872131
23	Shijiazhuang	0.481009	0.478092	0.462524	0.439574
24	Taiyuan	0.61132	0.568609	0.558776	0.617309
25	Tianjin	0.63171	0.624746	0.580419	0.618667
26	Wuhan	0.969384	0.743701	0.737345	0.701077
27	Urumqi	0.957401	0.944612	0.680446	0.97296
28	Xian	0.675965	0.826735	0.598937	0.652698
29	Xining	0.606469	0.610215	0.544589	0.570594
30	Yinchuan	0.768444	0.738936	0.658954	0.732169
31	Zhengzhou	0.755548	0.76361	0.791239	0.605025

**Table 2:** Thirty-one city two-stage efficiencies from 2013 to 2016.

NO.	DMU	2013 S-1	2013 S-2	2014 S-1	2014 S-2	2015 S-1	2015 S-2	2016 S-1	2016 S-2
1	Beijing	1	1	0.9841	0.859717	1	0.523655	0.967305	0.620516
2	Changchun	0.784014	0.777225	0.810363	0.683197	0.867712	0.440007	0.666735	0.722244
3	Changsha	0.867294	0.422157	0.854909	0.823818	0.847923	0.780553	0.862052	0.915606
4	Chengdu	0.631859	0.381234	0.599879	0.540916	0.666245	0.393688	0.588109	0.404624
5	Chongqing	0.57216	0.833457	0.577451	0.842549	0.577227	0.778049	0.58374	0.854677
6	Fuzhou	0.953513	1	0.612554	1	0.896525	1	0.882006	1
7	Guangzhou	1	1	1	1	1	1	1	1
8	Guiyang	0.430391	0.881465	0.464297	0.800737	0.48783	0.631223	0.496444	0.762751
9	Harbin	0.843138	0.523092	0.820257	0.468745	0.911042	0.304295	0.60593	0.619029
10	Haikou	0.932073	1	0.662616	1	0.894992	1	0.839084	1
11	Hangzhou	0.828935	0.421705	0.819238	0.561614	0.830777	0.405179	0.857406	0.588141
12	Hefei	0.750495	0.479733	0.748732	0.439018	0.734275	0.703888	0.901489	1
13	Huhehot	0.794014	0.749271	0.79885	0.768767	0.784616	0.579296	0.767966	0.754124
14	Jinan	0.69103	0.474712	0.681966	0.48622	0.648869	0.520022	1	1
15	Kunming	0.447209	0.924271	0.450665	0.824437	0.506021	0.870361	0.495605	0.849659
16	Lanzhou	0.512725	0.92661	0.437378	0.935135	0.448104	0.600829	0.457415	0.93038
17	Lhasa	1	1	1	1	1	1	1	1
18	Nanchang	0.849399	0.655427	0.83084	0.678777	0.794252	0.5873	0.662679	0.695209
19	Nanjing	0.865647	0.443562	0.822589	0.72271	0.880772	0.487604	0.918836	0.614334
20	Nanning	1	1	1	1	1	1	0.928314	1
21	Shanghai	1	1	1	1	1	1	1	1
22	Shenyang	0.677385	0.487561	0.668757	0.638463	0.651262	0.328558	1	0.761222
23	Shijiazhuang	0.420644	0.546837	0.420201	0.541625	0.381233	0.554448	0.388758	0.494223
24	Taiyuan	0.497082	0.745665	0.485602	0.6623	0.48296	0.64264	0.496031	0.76024
25	Tianjin	0.79577	0.49495	0.788973	0.489891	0.777944	0.420716	0.830746	0.451418
26	Wuhan	0.939486	1	0.759276	0.728561	0.760506	0.714807	0.806428	0.608009
27	Urumqi	0.91658	1	0.892165	1	0.594624	0.778365	0.947314	1
28	Xian	0.625065	0.730118	0.680201	1	0.615122	0.582851	0.559771	0.758082
29	Xining	0.497994	0.733845	0.4237	0.855227	0.420998	0.69296	0.443779	0.726317
30	Yinchuan	0.610989	0.961078	0.566618	0.954052	0.557347	0.775328	0.570696	0.932932
31	Zhengzhou	0.878029	0.645468	0.905744	0.638679	0.973174	0.63835	0.726674	0.497766

Note: S1 refers to Stage 1 in the DEA analysis; S2 refers to Stage 2 in the DEA analysis.

**Table 3:** Wilcoxon Test of efficiency for the high-income and upper middle–income countries.

	Total	Production Stage	Treatment Stage
2013	0.0590^*^	0.0064^**^	0.0094^**^
2014	0.2711	0.2083	0.2275
2015	0.0590^*^	0.0086^**^	0.0030^**^
2016	0.0569^*^	0.2264	0.1763

* less than 10% significant; ** less than 5% significant.

**Table 4:** First-stage input efficiencies.

No.	DMU	2013–2016 Average Labor	2013–2016 Average Asset	2013–2016 Average Energy Consumption
1	Beijing	0.9383	0.9780	0.9956
2	Changchun	0.8428	0.6555	0.8828
3	Changsha	0.9419	0.5077	0.6611
4	Chengdu	0.7700	0.6418	0.7700
5	Chongqing	0.5457	0.4480	0.7471
6	Fuzhou	0.9217	0.6527	0.9419
7	Guangzhou	1.0000	1.0000	1.0000
8	Guiyang	0.6463	0.5036	0.5556
9	Harbin	0.7143	0.6105	0.8928
10	Haikou	0.7574	0.9182	0.9447
11	Hangzhou	0.9201	0.6535	0.7757
12	Hefei	0.8974	0.5208	0.8974
13	Huhehot	0.8923	0.7088	0.6350
14	Jinan	0.8601	0.8368	0.6553
15	Kunming	0.6486	0.5393	0.6027
16	Lanzhou	0.6441	0.6225	0.3322
17	Lhasa	1.0000	1.0000	1.0000
18	Nanchang	0.8860	0.5537	0.8860
19	Nanjing	0.9455	0.5825	0.7513
20	Nanning	0.9069	0.8994	1.0000
21	Shanghai	1.0000	1.0000	1.0000
22	Shenyang	0.8612	0.5788	0.7322
23	Shijiazhuang	0.5828	0.5092	0.3962
24	Taiyuan	0.6742	0.6651	0.1782
25	Tianjin	0.9061	0.4395	0.6792
26	Wuhan	0.9094	0.5740	0.7818
27	Urumqi	0.8964	0.9026	0.8934
28	Xian	0.7718	0.5276	0.7718
29	Xining	0.6272	0.6054	0.3283
30	Yinchuan	0.7506	0.5442	0.3119
31	Zhengzhou	0.8945	0.6344	0.9369

**Table 5:** First-stage input efficiencies.

No.	DMU	2013–2016 Average GDP	2013–2016 Average CO_2_	2013–2016 Average AQI (Air Quality Index)
1	Beijing	0.9957	0.9935	0.9978
2	Changchun	0.9075	0.8822	0.6527
3	Changsha	0.9480	0.6615	0.8828
4	Chengdu	0.8428	0.7701	0.8041
5	Chongqing	0.8321	0.7445	0.9934
6	Fuzhou	0.7726	1.0000	0.9952
7	Guangzhou	1.0000	1.0000	0.9958
8	Guiyang	0.7931	0.5915	0.7348
9	Harbin	0.9182	0.8930	0.5992
10	Haikou	0.7959	0.9998	0.9938
11	Hangzhou	0.9312	0.7762	0.8889
12	Hefei	0.8563	0.8985	0.7460
13	Huhehot	0.9115	0.6353	0.5633
14	Jinan	0.8982	0.6545	0.7602
15	Kunming	0.7939	0.6027	0.9736
16	Lanzhou	0.7924	0.3301	0.5271
17	Lhasa	1.0000	0.9994	0.9957
18	Nanchang	0.9094	0.8865	0.8332
19	Nanjing	0.9513	0.7524	0.9186
20	Nanning	0.9617	0.9998	0.9961
21	Shanghai	1.0000	1.0000	0.9968
22	Shenyang	0.8988	0.7322	0.7841
23	Shijiazhuang	0.7727	0.4518	0.6126
24	Taiyuan	0.8028	0.1786	0.4634
25	Tianjin	0.9212	0.6792	0.7933
26	Wuhan	0.9254	0.7935	0.7364
27	Urumqi	0.8867	0.9183	0.8364
28	Xian	0.8440	0.8120	0.6197
29	Xining	0.7868	0.3272	0.5541
30	Yinchuan	0.8338	0.3122	0.5175
31	Zhengzhou	0.9481	0.9358	0.7081

**Table 6:** First-stage input efficiencies.

No.	DMU	2013–2016 Average Media	2013–2016 Average Health Expenditure	2013–2016 Average Birth Rate	2013–2016 Average Respiratory Diseases
1	Beijing	0.3643	0.551	0.90675	0.87775
2	Changchun	0.7965	0.4625	0.86225	0.7965
3	Changsha	0.6610	0.839	0.89275	0.839
4	Chengdu	0.6050	0.46	0.7805	0.605
5	Chongqing	0.9125	0.69775	0.9255	0.9125
6	Fuzhou	1.0000	1	1	1
7	Guangzhou	1.0000	1	1	1
8	Guiyang	0.8618	0.73475	0.90075	0.8715
9	Harbin	0.6468	0.46575	0.7975	0.64675
10	Haikou	1.0000	1	1	1
11	Hangzhou	0.5170	0.66425	0.801	0.66425
12	Hefei	0.6230	0.76225	0.86475	0.77825
13	Huhehot	0.8313	0.706	0.878	0.83475
14	Jinan	0.4268	0.74375	0.85425	0.761
15	Kunming	0.9415	0.551	0.948	0.9415
16	Lanzhou	0.3515	0.73225	0.9575	0.9385
17	Lhasa	1.0000	1	1	1
18	Nanchang	0.5108	0.53225	0.865	0.81325
19	Nanjing	0.5690	0.71575	0.82675	0.72425
20	Nanning	1.0000	1	1	1
21	Shanghai	1.0000	1	1	1
22	Shenyang	0.7003	0.63925	0.80625	0.64475
23	Shijiazhuang	0.4755	0.624	0.81675	0.7095
24	Taiyuan	0.5468	0.64575	0.882	0.84375
25	Tianjin	0.5703	0.2585	0.79575	0.65425
26	Wuhan	0.8633	0.71775	0.90075	0.86325
27	Urumqi	0.7778	0.97575	0.97975	0.97575
28	Xian	0.8383	0.613	0.90575	0.87175
29	Xining	0.3163	0.8015	0.90575	0.88175
30	Yinchuan	0.3618	0.9675	0.975	0.97025
31	Zhengzhou	0.3415	0.404	0.8515	0.785

**Table 7:** Media, CO_2,_ AQI, and respiratory diseases efficiency correlation test.

	CO_2_	AQI	Respiratory Diseases
2013Media	0.5861	0.4072	0.5932
2014Media	0.4275	0.3387	0.4252
2015Media	0.4384	0.6535	0.5751
2016Media	0.5631	0.5619	0.3697

**Table 8:** Technology gap ratio analysis.

NO	DMU	2013	2014	2015	2016
1	Beijing	1	0.920417	0.951156	0.905517
2	Changchun	0.799766	0.865208	0.626083	0.765738
3	Changsha	0.688145	0.876117	0.813482	0.888706
4	Chengdu	0.649096	0.66255	0.777481	0.7262
5	Chongqing	0.692683	0.700274	0.67162	0.709513
6	Fuzhou	0.976286	0.998914	1.001418	0.986605
7	Guangzhou	1	1	1	1
8	Guiyang	0.804812	0.789525	0.765699	0.783652
9	Harbin	0.78929	0.742931	0.661526	0.771179
10	Haikou	0.965631	0.891907	0.9715	0.934996
11	Hangzhou	0.892932	0.894409	0.914981	0.9587
12	Hefei	0.895494	0.881029	0.776732	0.948708
13	Huhehot	0.771201	0.783619	0.675695	0.761003
14	Jinan	0.845658	0.839612	0.801471	1
15	Kunming	0.742354	0.754756	0.790251	0.779946
16	Lanzhou	0.846304	0.818117	0.772737	0.777249
17	Lhasa	1	1	1	1
18	Nanchang	0.747725	0.751731	0.697362	0.703055
19	Nanjing	0.843292	0.887507	0.926665	0.952523
20	Nanning	1	1	1	0.963501
21	Shanghai	1	1	1	1
22	Shenyang	0.77126	0.724574	0.755926	0.872131
23	Shijiazhuang	0.76481	0.769609	0.718522	0.731075
24	Taiyuan	0.766194	0.758631	0.75676	0.718906
25	Tianjin	0.866574	0.975213	0.943439	0.952695
26	Wuhan	0.980431	0.955047	0.970918	0.997056
27	Urumqi	0.957401	0.944612	0.680446	0.97296
28	Xian	0.821765	0.875207	0.809363	0.767682
29	Xining	0.890846	0.81891	0.821108	0.801893
30	Yinchuan	0.831326	0.817149	0.794892	0.789536
31	Zhengzhou	0.755548	0.76361	0.791239	0.901562

**Table 9:** Technology gap between the production stage and the health management stage in each city.

NO	DMU	2013 S1	2013 S2	2014 S1	2014 S2	2015 S1	2015 S2	2016 S1	2016 S2
1	Beijing	1	1	0.9841	0.859717	1	0.894374	0.977258	0.829966
2	Changchun	0.784014	0.816048	0.810363	0.924697	0.867712	0.440007	0.666735	0.880661
3	Changsha	0.879721	0.524584	0.932426	0.823818	0.847923	0.780553	0.862052	0.915606
4	Chengdu	0.631859	0.655117	0.599879	0.730815	0.666245	0.924537	0.588109	0.917548
5	Chongqing	0.57216	0.833457	0.577451	0.842549	0.577227	0.778049	0.58374	0.854677
6	Fuzhou	0.953513	1	0.997512	1	1.002561	1	0.974467	1
7	Guangzhou	1	1	1	1	1	1	1	1
8	Guiyang	0.633033	0.994539	0.596955	1.026177	0.579653	1.010953	0.597173	1.01622
9	Harbin	0.853814	0.722546	0.820257	0.661141	0.915633	0.440075	0.608234	0.98388
10	Haikou	0.932073	1	0.791953	1	0.945958	1	0.880062	1
11	Hangzhou	1.007733	0.777175	0.970302	0.819937	0.970346	0.849596	0.961007	0.956052
12	Hefei	0.805091	1.00597	0.79557	0.985432	0.734275	0.823614	0.901489	1
13	Huhehot	0.794014	0.749271	0.79885	0.768767	0.784616	0.579296	0.767966	0.754124
14	Jinan	0.980467	0.721654	0.910867	0.768498	0.91115	0.701844	1	1
15	Kunming	0.548987	0.978743	0.570825	0.977019	0.626686	0.985638	0.608274	0.987527
16	Lanzhou	0.703492	1.011085	0.605101	1.079956	0.604982	0.983986	0.568089	1.046075
17	Lhasa	1	1	1	1	1	1	1	1
18	Nanchang	0.849399	0.655427	0.83084	0.678777	0.823516	0.5873	0.710809	0.695209
19	Nanjing	0.999461	0.698904	0.987955	0.797774	0.972082	0.876073	0.970163	0.935064
20	Nanning	1	1	1	1	1	1	0.928314	1
21	Shanghai	1	1	1	1	1	1	1	1
22	Shenyang	0.835419	0.70751	0.823663	0.638463	0.796233	0.70001	1	0.761222
23	Shijiazhuang	0.605896	0.962323	0.620055	0.953419	0.566882	0.901437	0.560729	0.952653
24	Taiyuan	0.58826	0.989451	0.556767	1.031572	0.576715	0.988437	0.518786	0.985778
25	Tianjin	0.986385	0.752208	1.000127	0.948332	1.013662	0.860202	1.045958	0.854936
26	Wuhan	0.961032	1	0.929586	0.981111	1.00099	0.941638	0.99833	0.995729
27	Urumqi	0.91658	1	0.892165	1	0.594624	0.778365	0.947314	1
28	Xian	0.72997	0.924013	0.762626	1	0.681047	0.97043	0.614017	0.957543
29	Xining	0.805349	0.979803	0.650721	1.007062	0.668876	0.991888	0.627061	1.015135
30	Yinchuan	0.697177	0.985967	0.635244	1.040927	0.633286	0.993166	0.621991	0.995286
31	Zhengzhou	0.878029	0.645468	0.905744	0.638679	0.973174	0.63835	0.990239	0.811945

**Table 10:** Wilcoxon Test of technology gap for the high-income and upper middle–income countries.

	Total	Production Stage	Treatment Stage
2013	0.0238**	0.0016^**^	0.0010^**^
2014	0.0787^*^	0.0453^**^	0.0929^*^
2015	0.0344^**^	0.0006^**^	0.0003^**^
2016	0.0169^**^	0.0065^**^	0.0199^**^

* less than 10% significant; ** less than 5% significant.
